# Axonal Computations

**DOI:** 10.3389/fncel.2019.00413

**Published:** 2019-09-18

**Authors:** Pepe Alcami, Ahmed El Hady

**Affiliations:** ^1^Division of Neurobiology, Department of Biology II, Ludwig-Maximilians-Universitaet Muenchen, Martinsried, Germany; ^2^Department of Behavioural Neurobiology, Max Planck Institute for Ornithology, Seewiesen, Germany; ^3^Princeton Neuroscience Institute, Princeton University, Princeton, NJ, United States; ^4^Howard Hughes Medical Institute, Princeton University, Princeton, NJ, United States

**Keywords:** analog-digital signaling, action potential generation, propagation, resistance, capacitance, myelin, axo-axonal coupling

## Abstract

Axons functionally link the somato-dendritic compartment to synaptic terminals. Structurally and functionally diverse, they accomplish a central role in determining the delays and reliability with which neuronal ensembles communicate. By combining their active and passive biophysical properties, they ensure a plethora of physiological computations. In this review, we revisit the biophysics of generation and propagation of electrical signals in the axon and their dynamics. We further place the computational abilities of axons in the context of intracellular and intercellular coupling. We discuss how, by means of sophisticated biophysical mechanisms, axons expand the repertoire of axonal computation, and thereby, of neural computation.

## 1. Introduction

Neurons are compartmentalized into input compartments formed by dendrites and somas, and an output compartment, the axon. However, in the current era, there is a widespread tendency to consider that the biophysics of single neurons do not matter to understand neuronal dynamics and behavior. Neurons are often treated as point processes with disregard of the complex biophysical machinery that they have evolved. Moreover, neuronal computations are assumed to be mostly performed by dendrites or at synapses (Südhof and Malenka, 2008; Stuart et al., 2016), and axons are reduced to simple, static, and reliable devices. However, a wealth of literature supports that this is not the case: axons form complex structures that ensure a variety of sophisticated functions and they are highly dynamic. Here, we aim to review evidence that axons perform complex computations which depend on a myriad of biophysical details and ensure the generation and propagation of neuronal outputs. We will not discuss biophysics of synaptic release, reviewed elsewhere (Südhof and Malenka, 2008).

**Figure 1:**
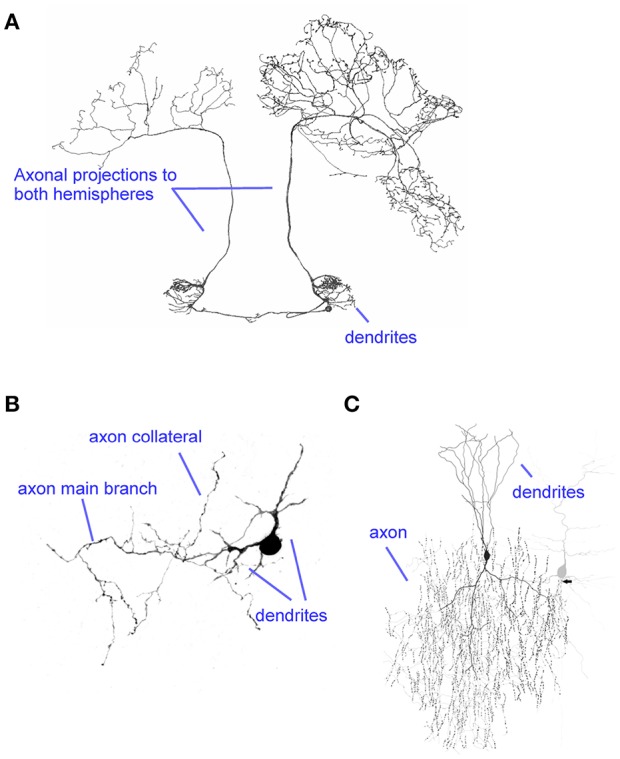
Diversity of axons. **(A)** The axon of the BAC cell in the honeybee projects onto the left and right hemispheres, targeting a large number of regions. Modified from Zwaka et al. (2016) (CC-BY). **(B)** A cerebellar rat stellate cell axon illustrates a small local axon. Modified from Alcami and Marty (2013). **(C)** The extensive arborization of a cortical chandelier cell targets the axon initial segment of many postsynaptic cells (example shown in gray). Modified from Wang et al. (2016) (CC-BY).

More than six decades after seminal discoveries in experimentally accessible invertebrate axons (Hodgkin and Huxley, 1952), axonal research has unraveled previously-unsuspected, rich and dynamical electrical signaling in axons, which consists of a hybrid of analog and digital signaling. Contrary to the giant invertebrate axons studied in the early days (Hodgkin and Huxley, 1952; Furshpan and Potter, 1959), most invertebrate and vertebrate axons are thin and present complex extended arborizations (e.g., Figure 1), making it difficult to record from them. However, successful electrophysiological recordings from axons and terminals have been performed (Hu and Jonas, 2014; Kawaguchi and Sakaba, 2015) and electron and optical microscopy have made it possible to deepen our understanding of fine axonal structures (Rash et al., 2016; D'Este et al., 2017). Moreover, the combination of new structural imaging techniques with electrophysiology has made it possible to study how structural changes at the sub-micrometer scale impact function (Chéreau et al., 2017).

We will first illustrate general signaling principles in axons, before delving into the generation and propagation of action potentials (APs) and their dynamical regulation. We will comment on the computational abilities enabled by different mechanisms throughout the article. Finally, we will position axons in the context of their interactions with other compartments and with each other, by virtue of axo-axonal coupling (Katz and Schmitt, 1940; Furshpan and Potter, 1959) and finally, indirectly via glial cells.

## 2. Review of Axonal Computations

### 2.1. General Principles: Signaling in Axons

#### 2.1.1. Of Axons and Brains

The propagation of electrical signals along axons controls the reliability and the timing with which neural networks communicate. The spatial extent of axonal trees introduces delays between the generation of an AP in a neuron and its arrival to the presynaptic site, where information is relayed to a postsynaptic cell. Thereby, the axonal propagation delay influences the temporal relationship of presynaptic and postsynaptic activity (Izhikevich et al., 2004).

The delays in AP propagation encode information (Seidl et al., 2010) and contribute to re-configuring neural circuits through plastic mechanisms such as spike timing dependent plasticity (Bi and Poo, 1998; Izhikevich et al., 2004). Each synapse is characterized by its own “critical window,” given by the delay between presynaptic and postsynaptic activity, to induce synaptic plasticity. The speed at which signals travel along axons can vary over four orders of magnitude, from tens of centimeters per second in thin unmyelinated axons to 100 m/s in giant myelinated fibers (Xu and Terakawa, 1999; Schmidt-Hieber et al., 2008); highlighting the ability of biophysical specializations of different axons to conduct APs at different speeds. In computational terms these specializations allow signals to travel at different speeds in neural circuits, diversifying and expanding the timescales at which neural computations take place. Moreover, they modulate the speed of information processing in neural circuits.

Axons have evolved different intricate geometries (Figure 1), revealing specific structure-function specializations. Constraints to axonal morphology include (1) spatial constraints: axons occupy large volumes of nervous systems, yet they require internal components necessary to their function such as mitochondria, which constrain their minimal functional size; (2) energetic requirements may have evolutionary exerted a selection pressure on axonal properties (Perge et al., 2009; Harris and Attwell, 2012); (3) efficiency of electrical information processing in relation to structure-function specializations. The last point on electrical information processing will be the main focus of the current review.

One first needs to define what a computation is in the context of neuronal information processing. We can state that the role of a neuron is to generate and transmit electrical signals. Ultimately, subthreshold signals and spikes propagate in axons, conveying a combined functional output message to the synaptic terminals (reviewed in Zbili and Debanne, 2019). These output signals need to first be generated by integrating input information received by soma and dendrites. Any modification of the input/output relation is to be considered a computation (Silver, 2010) since it contributes to the transformations that ultimately generate the functional message carried by axons. It is important to note that these modifications are inherently non-linear as they deviate from computations by a linear and static cable.

#### 2.1.2. Propagation of Electrical Signals in the Axon

Propagation of electrical signals in the axon results from a combination of specialized active and passive mechanisms. Active properties are shaped by neuronal voltage-gated ion channels recruited as a function of the dynamics of the membrane potential whereas passive properties are determined by the axonal membrane at rest and by axonal geometry.

**Figure 2:**
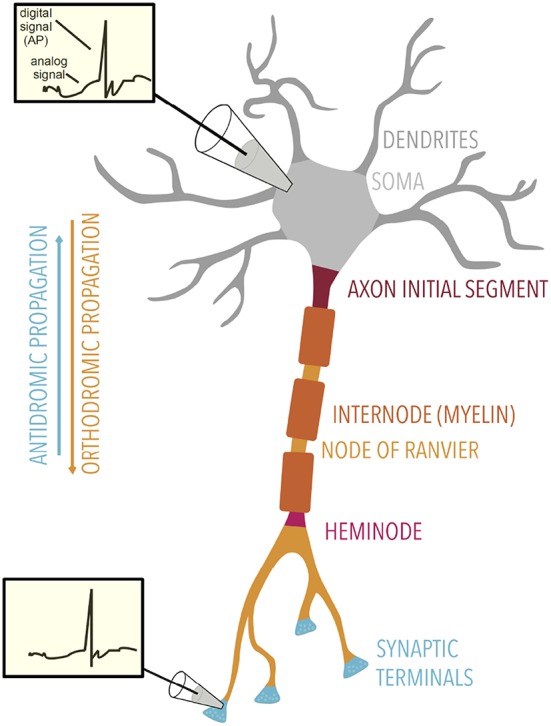
Overview of axonal electrical signaling. Axonal signaling is formed by a mixture of analog and digital signaling. Insets: membrane potential traces show both the analog and digital components, the former being attenuated with distance. Electrical signals can propagate orthodromically or antidromically. Specialized regions of the axon are labeled.

As increasingly appreciated, APs are not just an on/off switch, and electrical signaling in the axon should be regarded as a hybridization of analog and digital signaling (Shu et al., 2006) (Figure 2). Furthermore, APs are strongly regulated by the background analog activity provided by subthreshold postsynaptic events. For example, the inactivation of potassium *K*_*v*_1 channels in the axon initial segment broadens the axonal AP waveform and increases unitary excitatory postsynaptic potentials (EPSP) amplitude in layer V pyramidal neurons (Kole et al., 2007). Interestingly, Na_v_ channels have also been implicated in analog-digital signaling. The recovery from inactivation of axonal Na_v_ channels can increase AP amplitude, enhancing synaptic transmission (Rama et al., 2015). Note that some neurons do not encode information with APs but only with graded signals (graded potential neurons; Borst and Haag, 1996).

#### 2.1.3. Non-electrical Changes

Signaling in axons and neurons is usually regarded as purely electrical. However, electrical signals are accompanied by other biophysical changes in the neuronal membrane, some of which may further contribute to signaling. The AP is accompanied by changes in many biophysical properties such as temperature, mechanical membrane properties, and optical birefringence (Abbott et al., 1965; Cohen et al., 1970; Howarth, 1975; Howarth et al., 1975; Tasaki and Iwasa, 1982b; Tasaki and Byrne, 1992). For example the changes in the optical properties of the axon during propagation have been at the basis of the label free interferometric imaging of APs in *in-vitro* systems (Akkin et al., 2007; Oh et al., 2012; Batabyal et al., 2017).

Mechanical displacements associated with the AP have been measured in many experimental contexts (Hill et al., 1977; Iwasa and Tasaki, 1980; Tasaki and Iwasa, 1982a,b). These mechanical displacements can be regarded as propagating surface modes that are elicited via the large electrostatic force produced by the AP. In this regard, the AP is an electro-mechanical pulse (El Hady and Machta, 2015). Interestingly, converging evidence suggests that mechanics play a role in electrical signaling (Tyler, 2012). Moreover, there is mounting evidence that some voltage sensitive channels such as sodium and potassium channels are mechanically modulated locally and that many neurons express mechanically-activated channels (Schmidt and MacKinnon, 2008; Schmidt et al., 2012; Ranade et al., 2015). Interestingly, mechanically sensitive ion channels are present in the internodes of myelinated axons (Brohawn et al., 2019). Future research is needed to establish the functional relevance of non-electrical changes that accompany action potentials, including in particular the relevance of mechanical displacements.

### 2.2. Biophysics of Action Potential Generation

#### 2.2.1. A Brief Historical Perspective on the Action Potential

At the resting, non-excited state, mostly potassium channels are open and the resting potential is, as a consequence, close to the reversal potential for potassium, maintained around −70 mV. In 1939, Hodgkin and Huxley published the first trace of an AP recorded from the squid axon using an intracellular electrode where one can see a very clear overshoot. Following this in 1949, the proposal that sodium ions are the main mediator of AP generation was put forward by Hodgkin and Katz (1949). They studied the effect of systematically varying the concentration of sodium ions and measured its impact on the amplitude of the AP recorded. Subsequently, Keynes managed to show that nerve excitation leads to an increase in the transmembrane flow of sodium ions by tracing the movement of the radioactive isotope *Na*^24^ in repeatedly stimulated squid axons (Keynes, 1951). These seminal findings confirmed that sodium ions are the main contributors to AP generation.

#### 2.2.2. Variations of Action Potentials

APs are characterized by a width, a height and an overshoot magnitude (i.e., referring to the AP height beyond 0 mV). Despite sharing a general biophysical mechanism for initiation, the shape of APs varies in different types of neurons. For example, there are cells that exhibit very narrow APs of a few hundreds of microseconds in width, reflecting a fast spiking behavior, such as some GABAergic interneurons, Purkinje neurons which are GABAergic projection neurons, glutamatergic neurons of the subthalamic nucleus, and Medial nucleus of the trapezoid body (MNTB) cells in the superior olivary complex. CA1 pyramidal neurons in the hippocampus have, in contrast, a relatively wide AP and dopaminergic neurons have an even wider AP of up to 4 ms (reviewed in Bean, 2007).

Apart from the shape of the single spike, neurons can exhibit a diversity of firing patterns. For example, they can be bursting or non-bursting on one hand and they can be adapting or non-adapting on the other hand. Adaptation during a train of APs refers to the process by which AP properties, typically rate and amplitude, decrease within the spike train. There are many biophysical mechanisms by which spike frequency adaptation can happen. The most prominent mechanisms are an increase in outward current flowing through calcium-activated potassium channels and an increasing outward current produced by the electrogenic sodium-potassium pump (Powers et al., 1999). Moreover, there is also a substantial sodium channel inactivation induced by a long lasting depolarization (Sawczuk et al., 1997).

The diversity of AP shapes along with the firing patterns are a result of the different combinations of ion channels that the neuron expresses (reviewed in Marder and Goaillard, 2006). The expression of ion channels impacts the computational abilities of neurons (e.g., narrow action potentials and short after-hyperpolarizations allow cells to generate high firing rates). Narrow action potentials allow neurons to follow high frequency inputs with higher fidelity than cells with wide action potentials and long refractory periods. Remarkably, in addition to constraining bandwidth of information processing, different AP widths may convey different information content (Borst and Sakmann, 1999).

#### 2.2.3. The Axon Initial Segment

**Figure 3:**
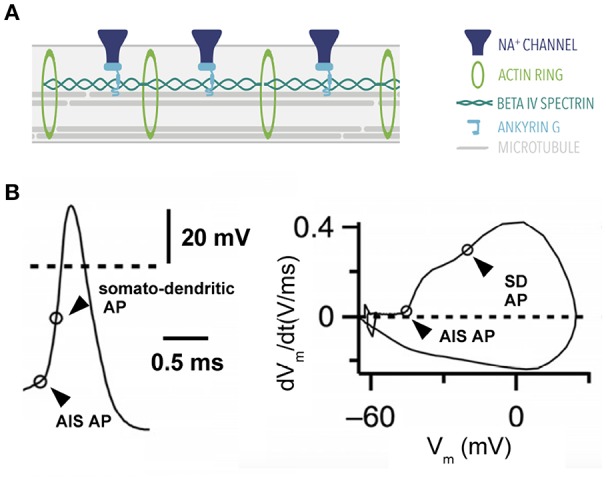
Action potential generation. **(A)** Ultrastructure of the axon initial segment. A highly-structured spatial organization characterizes proteins at the AIS. **(B)** Action potential of a stellate cell from the cochlear nucleus with two components: a fast rising phase of the action potential contributed by the AIS and a second phase contributed by the somatodendritic (SD) compartment. Right, phase plane plot of this cell. Modified from Yang et al. (2016) (CC-BY).

Before delving into the mechanistic details of AP generation, it is crucial to appreciate the complex anatomy of the Axon Initial Segment (AIS), the site of AP initiation (Figure 3A). The AIS is the main computational unit in axons, allowing them to integrate input signals and generate outputs (APs). Its distance from the soma can vary from 20–60 μm (Somogyu and Hamori, 1976; Sloper and Powell, 1979; Duflocq et al., 2011) to 120 μm (dopaminergic cells; Moubarak et al., 2019). The specific length of the AIS is variable across neurons as it has to adapt it to its own excitability properties. The excitability of the AIS is modulated by varying compositions of sodium and potassium channels clustered at the AIS.

The AIS contains a highly-specialized protein machinery that gives it a distinct character (reviewed in Leterrier, 2016). One such protein is Ankyrin G, a scaffolding protein that is also present at the nodes of Ranvier (Kordeli et al., 1995). Ankyrin G anchors voltage-gated channels such as voltage-gated sodium (*Na*_*v*_) and potassium channels (*K*_*v*_) to the membrane along with other adhesion molecules (Davis et al., 1996). Beta IV spectrin is another protein expressed in the axon initial segment. Beta IV spectrin's main function is to cluster sodium channels at the axon initial segment while simultaneously binding to the actin cytoskeleton (reviewed in Rasband, 2010; Figure 3A).

The AIS in mammalian neurons has been established as the site of the AP initiation following a series of seminal studies that began in the mid 1950s (Araki and Otani, 1955; Coombs et al., 1957; Fatt, 1957). The location of AP initiation has been further confirmed by combining precise electrophysiological measurements and imaging technologies. These allow to precisely identify the locus of AP initiation which was established to be in the distal part of the AIS, 20–40 μm from the soma (Palmer and Stuart, 2006; Kole et al., 2007; Meeks and Mennerick, 2007; Atherton et al., 2008; Foust et al., 2010; Palmer et al., 2010). The AIS is considered to be the site of AP initiation also because of several key properties. It contains a high sodium channel density. Furthermore, AIS sodium channels show a voltage dependence shifted to lower voltages, favoring their activation at less depolarized voltages than at the soma (Hu et al., 2009). Finally, the relatively large electrotonic distance of the AIS from the soma renders distal sodium influx more efficient in evoking a local membrane depolarization, compared to an AIS that would start at the soma. Note that electrophysiological recordings from invertebrates have shown that the initiation of the AP can happen at multiple locations acting in an independent manner (Calabrese and Kennedy, 1974; Meyrand et al., 1992; Maratou and Theophilidis, 2000), as will be discussed below. Along with its specialized protein machinery and acting as the site of AP initiation, the AIS also acts as a diffusion barrier between the somatodendritic and axonal compartments, filtering transport materials passing from soma to axon (Song et al., 2009; Brachet et al., 2010). Note however that the AIS length and its distance from the soma can be regulated in an activity-dependent manner (Grubb and Burrone, 2010; Kuba et al., 2014) and that in some neurons, AP initiation has been reported to occur at the first node of Ranvier (Clark et al., 2005; Lehnert et al., 2014), as will be developed later.

#### 2.2.4. The Sodium Ionic Dynamics and Action Potential Initiation

The extent to which the density of sodium channels is higher in the AIS and the contribution of this high density of sodium channels to AP initiation is a matter of active investigation. There is a consensus that sodium channel density is higher in the AIS but the order of magnitude is still unclear. Immunostaining of sodium channels consistently indicates that there is a higher channel density in the AIS of various neuron types (Wollner and Catterall, 1986; Boiko et al., 2003; Meeks and Mennerick, 2007). Lorincz and Nusser (2010) counted around 200 Na_v_ 1.6 sodium channels per square micrometer in the AIS of hippocampal pyramidal cells using electron microscopy. Given that the conductance of a single sodium channel is around 15 pS (Colbert and Johnston, 1996), one would expect a conductance density of about 3,000 pS per square micrometer. On the contrary, electrophysiological measurements from membrane patches pinpoint that sodium channel density is about 3–4 channels per square micrometer in the AIS, which is the same as the somatic density (Colbert and Johnston, 1996; Colbert and Pan, 2002). A similar conclusion was reached on the basis of recordings from blebs that form when cortical axons are cut in *in vitro* preparations. This might be due to the inability to draw AIS *Na*^+^ channels into the patch-clamp recording pipette due to their tight coupling to the actin cytoskeleton (Kole et al., 2008). In this article, the authors record a much larger sodium current after disruption of the actin cytoskeleton (Kole et al., 2008).

Kinetics of sodium currents underlying AP generation have been extensively studied. Hodgkin and Huxley have proposed that the activity of sodium channels can be fitted by *m*^3^ activation kinetics. Baranauskas and Martina (2006) found that sodium currents in three types of central neurons (prefrontal cortical cells, dentate gyrus granule cells and CA1 pyramidal cells) activate faster than predicted by Hodgkin-Huxley type kinetics following *m*^2^ activation kinetics. Moreover, it was found that the half activation voltage of voltage-gated sodium channels in layer 5 pyramidal neurons is 7–14 mV lower in the distal AIS compared to the soma and decreases further with increasing distance from the soma (Colbert and Pan, 2002; Hu et al., 2009). *Na*_*v*_1.6 channels, which are predominantly expressed in the axon initial segment and have a lower half activation voltage (Rush et al., 2005), are proposed to be primarily responsible for the initial slope of the AP. Furthermore, sodium channels at the AIS are more capable of producing a persistent sodium current (Stuart and Sakmann, 1995; Astman et al., 2006). The persistent sodium current has a significant influence on the AP threshold (Kole et al., 2008). Moreover, it is implicated in the generation of the AP afterdepolarization and it therefore contributes directly to the generation of high frequency AP bursts (Azouz et al., 1996).

Using sodium imaging to follow sodium influx in axon, soma and basal dendrites (Fleidervish et al., 2010), the authors suggest that the ratios of *Na*_*v*_ channel densities in these regions are approximately 3:1:0.3. Interestingly in another study by Lazarov et al. (2018), APs were initiated in the AIS, even when axonal *Na*_*v*_ channel density was reduced to about 10% in a beta IV spectrin mutant mouse. This experimental finding indicates to a great extent that AP initiation in the AIS does not require such a high local channel density. However in that study, the precision of AP timing was substantially compromised when axonal channel density was reduced. Likewise, the temporal accuracy of AP generation from MNTB cells decreases in a beta IV spectrin mutant mouse (Kopp-Scheinpflug and Tempel, 2015).

The aforementioned section highlights the complexity of sodium ion dynamics and begs for novel imaging modalities that allow tying ultra-fast dynamics with the axonal ultrastructures in order to get insights into the intricate biophysical mechanisms underlying the very first microseconds of AP initiation. The kinetics of sodium channel activation contribute to the speed at which neurons can generate action potentials. Thereby, they constitute a crucial factor in setting the computational speed of neural circuits. The existence of a diversity of sodium kinetics in different cell types likely allows neurons to be recruited at different speeds in different circuits.

#### 2.2.5. The Action Potential Rapidness

A simple but very informative way to study the properties of APs is to plot the time derivative of the voltage (dV/dt) versus the voltage. This is called “phase-plane plot.” The spike threshold can be easily visualized in such a representation, where it corresponds to the voltage at which dV/dt rises abruptly. Coombs et al. (1957) noticed that the main spike is preceded by a smaller earlier component (referred to as a “kink”). This component is interpreted as reflecting initiation of the spike in the initial segment of the axon. One can record such a component in somatic spikes in many central neurons, including neocortical pyramidal neurons which we will focus our discussion on here. As mentioned above, one of the most striking features of the AP recorded from cortical neurons has been the existence of a “kink” at the initiation of the AP. One can define the rapidness of AP onset in this cell as the slope of the phase-plane plot at dV/dt = 10 mV /ms. The AP rapidness can also be referred to as rate of voltage change. In Naundorf et al. (2006), the AP rapidness was measured from the cat visual cortical cells. AP rapidness varied between around 20–60 *ms*^−1^. It is important to mention that sharp, step-like onsets of APs have been recorded *in vivo* in many preparations (cat visual cortex: Azouz and Gray, 1999, and cat somatosensory cortex: in Yamamoto et al., 1990).

Several hypothesis have been proposed to explain the origin of the AP kink: (1) the backpropagation to the soma of a smoother AP generated at the AIS; (2) an abrupt opening of sodium channels due to the biophysics of neuronal compartmentalization; (3) a decreased membrane time constant due to the loading of the dendritic compartment; and (4) the cooperativity of sodium channels at the AIS.

The “lateral current hypothesis” states that the “kink” at spike onset reflects lateral current coming from the axon which becomes sharper through backpropagation from the initiation site to the soma while initiation is smooth at the initiation site (McCormick et al., 2007; Yu et al., 2008). In Yu et al. (2008), authors perform simultaneous recordings from axon blebs and soma, finding a smoother AP onset in the axon, additionally reproducing these results in a model. It is important to note that this study supporting the lateral current hypothesis was done in bleb recordings which are injured axons that may have undergone severe cytoskeletal reorganization (Spira et al., 2003) affecting sodium channels dynamics. This reorganization might alter the true dynamics of AP initiation.

Although the kink indeed might reflect the lateral current coming from the axon (Milescu et al., 2010), this hypothesis fails to account for the ability of neurons to follow 200–300 Hz frequency inputs. In order to account for this discrepancy, Brette (2013) proposed that the compartmentalization and the distance between the soma and the AIS leads to spike initiation sharpness. In his proposal, Brette suggests that the rapidness arises from the geometrical discontinuity between the soma and the AIS, rather than from the backpropagation of axonal APs. When sodium channels are placed in a thin axon, they open abruptly rather than gradually as a function of somatic voltage, as an all-or-none phenomenon.

Another proposal that takes into account the geometry of neurons is Eyal et al. (2014), in which the authors propose that increasing the dendritic membrane surface area (the dendritic impedance load) both enhances the AP onset in the axon and also shifts the cutoff frequency of the modulated membrane potential to higher frequencies. This “dendritic size effect” is the consequence of the decrease in the effective time constants of the neuron with increasing dendritic impedance load. The authors have shown this in a computational model of reconstructed layer 2/3 pyramidal neurons of humans and rats. The firing pattern at the axon is strongly shaped by the size of the dendritic tree. Authors predict that neurons with larger dendritic trees have a faster AP onset.

A last proposal to interpret AP onset rapidness is that sodium channels, which are assumed to be opening independently within the Hodgkin-Huxley framework, are gated cooperatively. The cooperativity model proposed that the half-activation voltage of the channels becomes dependent on the probability of the opening of the neighboring channels. These cooperative effects might happen mechanistically on very fast timescales either through a purely electrical, mechanical, or electro-mechanical coupling. Though there is no direct experimental test of the cooperativity of neuronal sodium channels at the AIS, it can theoretically account for the observed discrepancy between the sodium channel density and the very rapid rise of the AP at the site of initiation in the initial segment (Naundorf et al., 2006). It is important to note that cooperative gating has been previously observed in calcium, potassium, and HCN channels (Marx et al., 2001; Dekker and Yellen, 2006; Kim et al., 2014).

Apart from its mechanistic underpinnings, the fast rise of APs has attracted both experimental and theoretical approaches to study its functional implications on the biophysics of neuronal populations. Before detailing those functional implications, it is important here to go through a useful theoretical abstraction: a typical cortical neuron, embedded in a cortical network *in vivo*, receives about 10,000 synaptic inputs. Assuming that each of these synaptic inputs is active with a rate on the order of 1–10 Hz, incoming signals arrive at a rate of 10 kHz. As a result, the membrane voltage exhibits strong, temporally irregular fluctuations. To understand the computational capabilities of e.g., cortical circuits, it is essential to characterize single neuron computation under such realistic operating conditions. To control the activity of entire neuronal circuits while preserving their natural firing characteristics, it would be advantageous to introduce artificial input components mimicking intrinsically generated synaptic input under precise experimental control. In order to study the dynamical properties of cortical neurons, experimenters have mimicked synaptic bombardment *in vitro* by injecting stochastic inputs modeled as an Ornstein-Uhlenbeck process in which sinusoidal inputs are embedded (Destexhe et al., 2001; Tchumatchenko et al., 2010; Neef et al., 2013). This experimental setting has allowed the measurement of the dynamic gain of neurons, which means how much neurons attenuate their input in the frequency domain and how fast they are able to follow a rapidly-fluctuating input. This has led to the establishment of the ability of cortical neurons to follow high frequency inputs up to 200–300 Hz (Higgs et al., 2006; Higgs and Spain, 2009; Tchumatchenko et al., 2011). Moreover, there has been a series of theoretical studies exploring the dependence of encoding capacity on the active properties of the AP initiation (Fourcaud-Trocmé et al., 2003; Wei and Wolf, 2011; Huang et al., 2012).

#### 2.2.6. AP Trajectories Beyond the Rapidness of Initiation

Although we have mostly concentrated on the initial spike rapidness, note that the phase plot can display a variety of trajectories after the onset of the AP (Figure 3). These trajectories are determined by additional ion channels in conjunction with sodium channels. Potassium channels are typically responsible for the repolarizing phase of the AP. The relative temporal profiles of activation of sodium and potassium channels and their subunit composition determine the width of the AP (Lien and Jonas, 2003). Furthermore, the spatial location of ion channels also contributes to the shape of AP trajectories (Yang et al., 2016; Figure 3B). Interestingly, Kole et al. (2007) showed that APs become thinner during axonal propagation due to the specific expression of Kv1 channels in the axons of layer 5 pyramidal neurons. It has also been shown that the AP shape affects calcium currents and transmitter release [calix of Held: (Borst and Sakmann, 1999); dentate gyrus granule cells: (Geiger and Jonas, 2000); layer 5 pyramidal neurons: (Shu et al., 2006; Kole et al., 2007); CA3 pyramidal neurons (Bialowas et al., 2015; Rama et al., 2015); cerebellar interneurons: (Rowan et al., 2016); cerebellar Purkinje cells: (Kawaguchi and Sakaba, 2015)]. Therefore, the AP shape beyond the initial spike rapidness provides an extra dimension for information encoding on a variety of timescales.

#### 2.2.7. The Action Potential at Nodes of Ranvier

Although the AP is generated at the AIS, the nodes of Ranvier contain a machinery to regenerate the AP. It is important to note that there are striking similarities between axon initial segment and nodes of Ranvier. A great deal of the protein machinery in the axon initial segment is also present in the nodes of Ranvier where the regeneration of the AP is performed. Ankyrin G, the major scaffolding protein in the AIS and the ring-like arrangement of actin and beta IV spectrin are also found in the nodes of Ranvier (D'Este et al., 2017). Nodes of Ranvier are distributed spatially along the axon to guarantee the faithful propagation of the AP. In addition, the first node of Ranvier was found to be crucial for high bandwidth bursting activity in neocortical layer 5 pyramidal neurons (Kole, 2011). In that study, the author shows that nodal persistent sodium currents at the first node of Ranvier hyperpolarize AP threshold and amplify the afterdepolarization. The study opens up the space for a computational role of the first node of Ranvier beyond the regeneration of the propagating AP. The first node faithfully follows spike frequencies with a approximately 100 μs delay (Khaliq and Raman, 2006; Palmer and Stuart, 2006; Foust et al., 2010; Palmer et al., 2010). This had led to the speculation that the nodes of Ranvier, and in particular the first node, may have an active computational role in modulating the AP initiation itself.

It is worth noting that nodes of Ranvier also express *Na*_*V*_ 1.6, the same sodium channel subtype that is expressed in the AIS. It is even more striking that the sodium channels at the nodes also undergo the developmental changes from *Na*_*V*_ 1.2 to *Na*_*V*_ 1.6 during postnatal development, following a similar developmental trajectory as those found in the AIS (Rios et al., 2003). *Na*_*V*_ is highly clustered at the nodes of Ranvier in the order of 1,200 channels per micrometers square (Rosenbluth, 1976), while internodes contain 20–25 channels per micrometers square (Ritchie and Rogart, 1977). This very high density ensures high-fidelity regeneration of the AP. Given the close proximity of the first node to the cell body and high density of *Na*_*V*_ channels, it has been postulated that, in addition to securing propagation, it could potentially generate the AP (Colbert and Pan, 2002; Clark et al., 2005; Lehnert et al., 2014). Although this might be happening, the overwhelming evidence favors that the AP initiation is happening at the AIS.

#### 2.2.8. Ectopic Spiking

AP initiation at the AIS and its subsequent orthodromic propagation have been extensively investigated. However, a number of studies demonstrates that distally generated spikes or “ectopic” APs co-exist in invertebrate and vertebrate neurons (Mulloney and Selverston, 1972; Maranto and Calabrese, 1984; Meyrand et al., 1992; Sheffield et al., 2010; Dugladze et al., 2012; Lehnert et al., 2014). Marked differences in somatic AP recordings are observed when APs are generated at the AIS or in a distal part of the axon. In particular, APs have more negative thresholds when APs are generated in distal parts of the axon. This is likely due to the fact that they do not show the strong coupling of soma to AIS which is responsible for the inactivation of sodium channels at the AIS and for a more depolarized AP generation threshold. Interestingly, given the different potential at which ectopic spikes are generated, the conductances activated during AP generation may differ between distal and proximal APs (Meyrand et al., 1992).

In invertebrates, numerous examples of ectopic spikes and of their functional relevance have been described (Mulloney and Selverston, 1972; Maranto and Calabrese, 1984; Meyrand et al., 1992). Ectopic spikes in neurons that target the hearts of the leech have been proposed to control the heart firing frequency (Maranto and Calabrese, 1984). In the somatogastric ganglion of the crab, ectopic spikes are typically observed in the lateral gastric motor neuron only when the muscles remain attached to the preparation, ectopic spikes being induced by motor contraction (Meyrand et al., 1992). Remarkably, ectopic spikes in Meyrand et al. (1992) fail to depolarize terminals onto interneurons located close to the soma, whereas they efficiently excite a distal postsynaptic target, the muscle. Thus, orthodromically-propagating spikes generated close to the soma and antidromically-propagating spikes generated distally co-exist and they can reach synapses that target different postsynaptic neurons.

Ectopic spikes have also been observed in vertebrates. In principal cells from CA3 area in the hippocampus during rhythmic activity in the gamma range (Dugladze et al., 2012), axons fire APs at five times the firing frequency detected at the soma. This is due to the activity of a specific interneuron, the axo-axonic cell, that inhibits the initial segment, thereby avoiding the backpropagation of axonal spikes to the soma. Another noteworthy case of co-ocurrence of AIS and ectopic spikes has been reported in Lehnert et al. (2014) in auditory medial superior olive (MSO) neurons. A realistic model of MSO neurons which takes into account their axonal structure and ion channel composition generates APs at both the AIS and the first node of Ranvier. Additionally, under certain pathological conditions, e.g., in epilepsy, hyperexcitable axons have been reported to generate ectopic spikes in the hippocampus (Stasheff et al., 1993). To conclude, we would like to emphasize that ectopic spikes occur in some neurons because of their multiple action potential generation sites, that is, specialized regions that act as action—potential generating computational units.

We would like to make at this point a clarification: although “action potential” and “spike” are two terms used to refer to the sodium AP generated at the axon initial segment, the term spike seems preferentially used in the literature to refer to spikes that can differ in their location (dendritic or ectopic spikes) and in their underlying ionic mechanism (e.g., calcium spike, see below).

#### 2.2.9. Beyond the Sodium Spike

The spike initiated through sodium influx is not the only spike that can propagate in the axon. In some axons, there are spikes that are generated through the influx of calcium. In the giant axon of the jellyfish Aglantha digitale (order Hydromedusae), both sodium and calcium spikes propagate in the axon (Mackie and Meech, 1985). Sodium-dependent spikes are responsible for fast swimming and calcium spikes mediate slow swimming.

In vertebrates, calcium spikes are typically restricted to the dendritic compartment where there is a calcium spike generation mechanism. The biophysical mechanisms contributing to the dendritic spikes initiation in the distal apical trunk and proximal tuft of hippocampal CA1 pyramidal neurons (Gasparini et al., 2004) are dendritic sodium and potassium channels that set AP shape and propagation properties, the highly synchronized inputs, their spatial clustering and the activation of NMDA receptors. In the olfactory bulb mitral cells and in hippocampal and cortical pyramidal cells, dendritic spikes can trigger one or more axonal APs (Stuart et al., 1997; Golding and Spruston, 1998; Larkum et al., 1999, 2001; Chen et al., 2002; Ariav et al., 2003). In addition, backpropagating axonal APs can themselves promote dendritic spikes, a reciprocal interaction that can lead to a burst of axonal APs (Pinsky and Rinzel, 1994; Mainen and Sejnowski, 1996; Larkum et al., 1999, 2001; Doiron et al., 2002).

### 2.3. Biophysics of Action Potential Propagation

#### 2.3.1. An Equivalent Electrical Circuit for Axons

**Figure 4:**
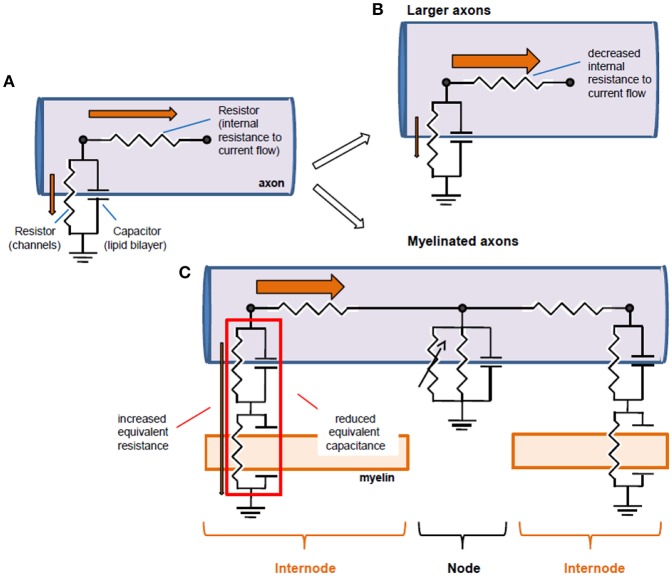
Equivalent circuits of axons and structural biophysical specializations that improve conduction. **(A)** An axon can be reduced to an equivalent electrical circuit involving an axial resistor (parallel to the membrane) conveyed by the intracellular medium of the axon (axoplasm), and a parallel “RC circuit” formed by the membrane resistance and the membrane capacitance. **(B)** Conduction can be improved by increasing axon diameter, thereby decreasing longitudinal resistance to current flow. This allows for a larger current flow in the axon relative to the membrane resistor, and thereby a larger space constant and faster conduction. **(C)** An alternative circuit modification occurs with myelination: additional capacitances conveyed by myelin in series with axonal capacitance reduce the effective capacitance and additional resistance increase the effective resistance, increasing propagation speed and space constant, respectively. Note that the battery associated to the membrane has been omitted for simplification purposes. Orange arrows represent the current flow, and their thickness is indicative of the relative current flow in the axoplasm and in the radial direction out of the axon.

The passive properties of axons can be modeled by an equivalent electrical circuit (Figure 4A). The axonal membrane can be reduced to two circuit elements: the lipid bilayer, modeled by a capacitor and ion channels, by a resistor. The resistance to axial current flow can be modeled by an additional resistance.

How fast signals propagate is critically controlled by the capacitance. Electrical currents need to first charge the membrane capacitance, that opposes the flow of electric current, before electrically-charged membranes can undergo voltage changes. Capacitances are proportional to the membrane capacity (capacitance per surface area) and to the membrane surface area. The capacity of neuronal membranes is in the order of 1 μF per *cm*^2^ (Gentet et al., 2000). Capacitance measurements from small axons are in the range of tens of picoFahrads (pF) (Mejia-Gervacio et al., 2007). Capacitances of larger neurons (i.e., strongly-ramifying interneurons and projection neurons) are therefore expected to be in the range of hundreds of pF to nF. Remarkably, dynamic changes in membrane capacitance are suggested by activity-dependent changes in the size of axons (e.g., Chéreau et al., 2017). However, little is known about dynamical changes in capacitance due to temperature, lipid composition, and whether these may significantly impact the propagation of electrical signals along the axon.

The membrane resistance plays a major role in controlling how far signals spread in space along the axon before membrane potential changes become imperceptible. This is typically measured by the space constant, defined as the distance over which the membrane potential decays to 37% of its initial value. The more open channels are available at the membrane, the more the axial current is attenuated in space along the axon due to current leakage through membrane channels. Remarkably, the membrane resistance can change (reviewed in Debanne et al., 2019), providing a potential source of variation in the conduction of electrical signals along axons.

**Figure 5:**
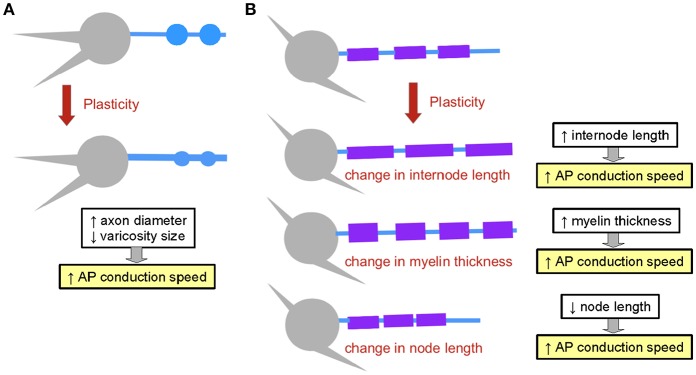
Structural plasticity mechanisms affecting the propagation of electrical signals. **(A)** Axon structural plasticity. Axons can change their diameter and bouton size. **(B)** Myelin structural plasticity can involve changes in internode length, in myelin thickness or in node length. The impact of each mechanism on AP propagation speed is outlined.

The axonal axial resistance to current flow results from the combination of the resistivity of the axoplasm (that is, the cytoplasm of the axon), given in Ohms*cm, and the diameter of the cylinder. Axial resistivity values are in the range of approximately 100 Ω.cm (Carpenter et al., 1975; Cole, 1975). Computational models of the calyx of Held suggest that the latency and amplitude of signals propagating between release sites is highly sensitive to changes in axial resistivity, and that these changes may have a large impact on synaptic release (Spirou et al., 2008). However, changes in resistivity have not been reported so far experimentally. The axon diameter can vary in three orders of magnitude, from hundred nanometers to hundreds of micrometers in diameter. Giant fibers found in invertebrates are an example of specialized large-diameter structures that propagate electrical signals with high speed over long distances (in Hodgkin and Huxley, 1952; Xu and Terakawa, 1999; Figure 4B). The larger the diameter of the axon, the faster the electrical signal propagates. Taking into account cable theoretic considerations, the propagation speed increases with the square root of the diameter. Remarkably, the axon diameter, as well as the diameter of synaptic boutons, are not static properties of axons. In fact, axon diameter has been shown to be regulated in hippocampal principal cells in an activity-dependent manner. Plasticity protocols induced changes in axonal diameter which were accompanied by significant changes in AP conduction velocity along CA3 pyramidal cell axons (Chéreau et al., 2017; Figure 5A).

#### 2.3.2. Propagation of Subthreshold Signals

Subthreshold signals propagate passively in axons, reaching synaptic terminals, where they influence spike-evoked synaptic release (Alle and Geiger, 2006). Subthreshold membrane fluctuations consist of synaptic events, typically in the range of 100 s of μVs to mVs, which in cortical neurons approximate highly stochastic background dynamics *in vivo* (Rudolph and Destexhe, 2003). Their propagation, referred to as analog, in comparison to the digital nature of the AP, modulates the efficiency of APs to induce neurotransmitter release in synaptic terminals (Shu et al., 2006). Given that subthreshold signals are not regenerated along the axon, analog signaling is more prominent in proximal portions of the axon. The presence of combined analog and digital signals in axons has been termed hybrid analog-digital signaling (reviewed in Zbili and Debanne, 2019).

Of particular interest is that the propagation of slow analog subthreshold signals does not only reach chemical synapses. Subthreshold signals also reach axonal electrical synapses, at which the signal is expected to be conveyed with high efficiency to the postsynaptic site due to the continuous and low-pass filtering properties of electrical transmission (reviewed in Alcami and Pereda, 2019).

The co-existence of subthreshold signals and suprathreshold signals (APs) illustrate two co-existing signaling modalities and computations in axons (reviewed in Zbili and Debanne, 2019).

#### 2.3.3. Reliability of Propagation and Action Potential Failures

Before we consider how signals propagate along axons, let us discuss the reliability of propagation. In small axons, modeling shows that APs can propagate with a large variability in their kinetics and amplitude due to channel noise (Neishabouri and Faisal, 2014). Furthermore, APs do not always efficiently propagate. In fact, failures in AP propagation are observed in many neuron types at high firing frequencies (Krnjevic and Miledi, 1959; Grossman et al., 1979; Monsivais et al., 2005).

Although geometry can generate failures (e.g., at branch points, see next section), failures typically involve active mechanisms during repetitive activation of axons as observed in a number of vertebrate and invertebrate preparations (Krnjevic and Miledi, 1959; Mar and Drapeau, 1996; Monsivais et al., 2005). Repetitive activity typically leads to extracellular potassium accumulation and subsequently to the depolarization of the axon, inducing failures of conduction by inactivating sodium channels (Grossman et al., 1979). In other cases however, repetitive activation induces AP propagation failures via hyperpolarization of the membrane (Mar and Drapeau, 1996). Additionally, computational modeling suggests that axonal gap junctions, by leaking current to coupled axons, may induce propagation failures in thin axons (Hull et al., 2015).

Note that propagation failures likely affect only a fraction of APs at physiological firing rates, and that most APs succeed in propagating. Remarkably, auditory axons are able to transmit action potentials at very high firing rates up to 1 kHz without failures (Kim et al., 2013).

Interestingly, changes in AP failure can also occur in response to membrane fluctuations, e.g., in response to synaptic inputs. Indeed, specific potassium channels of the type *I*_*A*_ underlie a hyperpolarization-mediated conduction block in hippocampal pyramidal cells (Debanne et al., 1997). Remarkably, the activation and de-inactivation kinetics of *I*_*A*_ allow for a history-dependent conduction block of APs.

Propagation failures, that occur under specific conditions or under high frequency firing, induce a mismatch between action potentials at the AIS and at more distal portions of the axon where synapses are located. Interestingly, this mismatch may impact the ability of neurons to drive synapses at high frequencies, even when AIS spiking could be driven at such high frequencies. Thereby, propagation failures represent under specific conditions, a limitation to the reliable propagation of action potentials.

#### 2.3.4. More Than Linear Cables: Impact of Axonal Branching and Inhomogeneities

Axons are typically formed by complex trees and spatial heterogeneities. These include branching points, varicosities (local enlargements of the axon containing the release machinery of chemical presynaptic sites) and large structures specialized in the interaction with other cells. Examples of such structures are the “basket” formed by a specific type of interneuron, basket cells, around principal cells in many brain regions; the glomerular collateral of the climbing fiber in the cerebellum; the “pinceau” structure surrounding cerebellar Purkinje cells (Palay and Chan-Palay, 2012) or the calyx of Held in the auditory system.

Changes in axon diameter at varicosities or branching points are characterized by an impedance mismatch, that is, a need of a larger current to flow in one of the two directions to electrically load axonal branches, provoking changes in AP propagation speed (Goldstein and Rall, 1974; Manor et al., 1991). Due to this impedance mismatch, when APs need to load a larger impedance as they propagate from one mother branch into two daughter branches, their propagation will be delayed relative to the speed that they would have had if no branching was present.

In the most extreme case, the electrical signal fails to load one or two of the branches, resulting in AP propagation failure. Propagation failures have been shown to occur in branches of a number of neurons (Yau, 1976; Grossman et al., 1979; Gu et al., 1991). An interesting example is provided by the medial pressure sensory neuron in the leech, where the failure of APs to propagate can differentially affect postsynaptic cells contacted by distinct presynaptic branches (Gu et al., 1991).

Finally, branching points can also provoke a surprising effect: APs can be slowed down in the ms range, up to a level that allows the mother branch to overcome the refractory period for AP generation. As a consequence, the AP can “reflect” (that is, travel backwards), increasing synaptic release at synapses present in the branch where the AP reflects (Baccus, 1998; Baccus et al., 2000).

The impact of axonal morphologies on AP propagation is likely to be relevant in the complex axonal ramification patterns of many neurons, including vertebrate interneurons (Ofer et al., 2019). The usage of voltage sensitive-dyes that track membrane voltage with sub-millisecond precision (Palmer and Stuart, 2006) should allow following large portions of axons both *in vitro* and *in vivo*, and characterize the propagation of APs along complex axonal structures.

At this point, we would like to comment on the relevance of the physiological phenomena constrained by the morphology of axons discussed above to non-linear processing by neurons, and therefore on their computational abilities. They introduce non-linear transformations in specific axonal compartments. Thereby, they allow for a differential encoding in different regions of the axonal tree, modulating the functional impact of APs onto different post-synaptic targets. In other words, there is not one axon, but multiple compartments in one axon. The details of AP propagation in these compartments have been explored by computational modeling, creating a detailed knowledge of how electrical activity flows in single cells (Peterson et al., 2011; Xylouris et al., 2011; Agudelo-Toro and Neef, 2013)

#### 2.3.5. Biophysical Properties of Myelinated Fibers

Many vertebrate and some invertebrate fibers are myelinated, a specialization endowed by the glial ensheathment of axons that appeared several times during evolution (Castelfranco and Hartline, 2015). Myelin, formed by compact lipidic layers produced by the membranes of glial cells (oligodendrocytes in the central nervous system and Schwann cells in the peripheral nervous system), strongly impacts the propagation of electrical signals.

In his seminal study, Lillie (1925) wrapped an iron wire placed in an acidic solution with an insulating glass cylinder. This increased the speed of propagation of electrical signals along the wire, which he postulated to occur in myelinated fibers. His prediction was confirmed decades later in axons by recording electrical signals at nodes, leading to the concept of the “saltatory” nature of transmission of electrical signals in myelinated fibers (Huxley and Stämpfli, 1949). Saltatory comes from the latin verb “saltare” (to jump), an analogy describing the very fast propagation of electrical signals between nodes of Ranvier.

Myelin modifies the electrical circuit that models the passive properties of axons described above by adding compact membrane layers in series with the axonal membrane. As a consequence, myelin increases the effective radial resistance and decreases the effective capacitance of the axon (Figure 4C). These two effects are due to the different properties of series resistors and series capacitors added to the circuit: series resistors sum their resistances whereas the inverse of capacitances from capacitors in series sums. The increase in effective membrane resistance (Bakiri et al., 2011) and the decrease in effective membrane capacitance by myelin have two major consequences. On the one hand, the increase in the effective axonal resistance by myelin increases the length constant. On the other hand, as a consequence of the decrease in the effective axonal capacitance, the time required to effectively load axons decreases, dramatically accelerating the propagation of electrical signals. This speeding of electrical propagation by a reduced effective capacitance underlies saltatory conduction between nodes of Ranvier (Huxley and Stämpfli, 1949; Castelfranco and Hartline, 2015). Therefore, adding myelin to an axon allows it to overcome the passive constraints that limit its computational abilities, including the speed of propagation and the distance of effective propagation of electrical signals. It additionally impacts the reliability and the jitter at which APs propagate, as will be discussed below.

#### 2.3.6. Geometry of Axons and Myelin

Theoretical studies have shown that specific myelination parameters maximize the space constant and the conduction velocity of electrical signals in axons. In particular, the ratio of the axonal diameter d to the fiber diameter D (the summed diameter of axon and myelin sheath), defined as the “g-ratio,” controls the conduction of electrical signals. Rushton (1951) developed a biophysical formalism to model current flow in a myelinated axon. He deduced the relation between the ratio l/D (internode length l over D) and the g-ratio. He further demonstrated that the ratio l/D is maximal when g = 0.6, a value also found analytically to maximize the space constant. In an independent approach, Deutsch (1969) mathematically derived the geometry of axonal and myelin properties that maximize, this time, conduction velocity. He deduced that the propagation velocity is inversely proportional to the RC time constant given by the internal resistance of the axon R and the capacitance of the membrane C. Maximizing conduction speed requires minimizing the time constant of the circuit. This resulted in the same geometrical properties as those derived by Rushton: a g-ratio of 0.6. Additional modeling studies including a more complete description of myelinated fibers converged to similar conclusions (Goldman and Albus, 1968). Therefore, due to the biophysical constraints posed by the thickness of myelin and axonal size, both conduction speed and efficient spatial propagation of electrical signals are maximized by specific axonal and myelin geometries. It is noteworthy that the geometry of axons and myelin does not only control AP propagation speed (Rushton, 1951; Deutsch, 1969) but also AP temporal jitter (Kim et al., 2013).

It seems difficult to imagine that neurons have fine-tuned their dendritic computations in a cell-type specific manner (Stuart et al., 2016), but that axons would, on the contrary, be invariant and homogeneously optimizing speed by their geometry. Additionally, we know that nervous systems adapt and fine tune a plethora of properties to accomplish specific functions (Marder and Taylor, 2011). Axons indeed adjust different parameters which impact the speed of propagation of electrical signals (Seidl et al., 2010; Ford et al., 2015; Arancibia-Carcamo et al., 2017) to obtain different speeds of computation.

An illustrative example of how the geometry of axons and myelin is tuned to adjust AP propagation speed, deviating from a g-ratio of 0.6, is provided by the axon properties which encode spatial location in the avian brain (Seidl et al., 2010). The longer contralateral fibers have larger-diameter axons and longer internodal distances, compensating in this manner for an otherwise larger conduction delay relative to the shorter fibers on the ipsilateral side. In this manner, axons ensure a coincident arrival of contralateral and ipsilateral signals to the synaptic terminals. As a consequence, post-synaptic cells can act as coincidence detectors, a key computational feature that allows neural circuits to locate sounds. Another interesting example is provided by fibers specialized in carrying information for low-frequency sounds, which show larger diameters than those carrying high-frequency sounds, but also shorter internodes. In doing so, these fibers deviate from the classical dependence of both variables established by Rushton. This specialization is proposed to ensure a proper function of the circuit (Ford et al., 2015). Moreover, internode length and node diameter show graded properties as they approach the terminal in the auditory granular bushy cell axon, implementing an efficient invasion of the axon terminal by APs (Ford et al., 2015).

We have seen that a large number of structural myelination parameters act in concert to control the speed of AP propagation (Goldman and Albus, 1968). It is noteworthy to mention that myelin has introduced new degrees of freedom in the regulation of AP speed: speed depends on distance between nodes of Ranvier, on node length, node composition, and thickness of myelin (Rushton, 1951; Wu et al., 2012; Ford et al., 2015; Arancibia-Carcamo et al., 2017). These parameters can be modulated independently or in combination, thereby increasing the number of available mechanisms by which nervous systems tune the axonal propagation of electrical signals. Furthermore, dynamical changes in axons and myelin (Sampaio-Baptista et al., 2013; McKenzie et al., 2014; Fields, 2015; Sinclair et al., 2017) suggest that nervous systems dynamically adjust the properties of their fibers to achieve the specific behaviors that they control. Adding myelin has allowed axonal conduction speed to be regulated by additional mechanisms other than their diameter. Indeed new adjustable parameters appeared evolutionary with myelination (e.g., myelin thickness, node length, etc.) to dynamically fine tune the computational speed of neural circuits.

#### 2.3.7. Active Contributions to Variations of Action Potentials Along the Axon

Each active AP regeneration site along the axon can potentially generate AP variants due to their specific state and composition of ion channels and membrane potential. In particular, myelinated fibers are characterized by the presence of non-myelinated sections, which concentrate the machinery to generate APs. These include nodes, the heminode (last unmyelinated portion before the terminal) and synaptic terminals. Remarkably, AP shape can be modified along the axon by the active channels present in these locations.

An example of local variations in AP shape through active mechanisms is found at presynaptic mossy fiber terminals (present in the axon of dentate gyrus granule cells), where local potassium channels modulate spike shape (Alle et al., 2011). Another example is provided by the exclusion of sodium channels from the terminals in the calix of Held, while being concentrated at the heminode. Such compartmental specialization has been suggested to produce APs with a shorter width in the calix (Leão et al., 2005). At the Purkinje cell nodes of Ranvier, the activation of calcium-dependent potassium channels repolarizes membranes, de-inactivating sodium channels which can then generate fast frequency spikes, and in this manner prevent AP failure at high frequencies (Gründemann and Clark, 2015). Additionally, changes in membrane voltage in the axon induced by active mechanisms can impact the efficiency of AP propagation. For example, in the leech touch cells, adaptation in response to repetitive AP firing consists of a hyperpolarization, resulting in the blockade of AP propagation (Van Essen, 1973).

We previously described how actively-generated signals can differently be passed on by two bifurcating branches, illustrating how passive properties can filter actively-generated signals (Gu et al., 1991). Remarkably, active properties of axonal branches can prevent failures by potentiating signals in specific branches in cultured hippocampal neurons (Cho et al., 2017). Failures that would occur as a consequence of the passive filtering of electrical signals can be prevented in a branch-specific manner by the presence of the sodium channel subunit NavβII, which potentiates AP propagation. Thus, the combination of passive and active properties fine tunes the computational properties of axonal branches.

#### 2.3.8. The Geometries of Axons and Myelin Are Plastic

Geometrical properties of both axons and myelin have been shown to be highly plastic. Indeed, the diameter of axons can change as a function of neuronal activity (Chéreau et al., 2017; Sinclair et al., 2017). Furthermore, the understanding of the mechanisms of myelination has revealed that properties of nodes and internodes are subject to plasticity (Young et al., 2013; reviewed in Kaller et al., 2017). Interestingly, major macroscopic changes in myelination occur in response to training paradigms in mice, allowing the acquisition of motor skills (Sampaio-Baptista et al., 2013; McKenzie et al., 2014; Xiao et al., 2016), and models of injury induce strong remodeling of myelination patterns in the auditory brainstem (Sinclair et al., 2017). Changes in myelination can involve several mechanisms: changes in internode length (Etxeberria et al., 2016), in myelin thickness (Sinclair et al., 2017) and in node length (suggested in Arancibia-Carcamo et al., 2017; Figure 5B). Remarkably, plasticity of myelination allows circuits to adjust their speed of computation, adding computational flexibility to neural networks.

#### 2.3.9. Nanostructures With Unknown Function

The evolutionary appearance of myelin has led to new specialized microstructures or microdomains (namely nodes, paranodes, juxtaparanodes, heminodes). Electron microscopy and recently superresolution imaging have revealed additional structures whose contribution to axonal computation remains mysterious. These structures can be observed at the nanometer scale in a highly regular spatial organization (D'Este et al., 2017), rising the question of the function of these periodic structures. For example, d'Este et al. show that the voltage-dependent potassium channel subunit Kv1.2 channels, found at the juxtaparanodes, correlates in space with the underlying actin cytoskeleton. An additional structure is the Schmidt-Lanterman incisure, a spiral cytoplasmic expansion from the outer tongue of myelin to the inner tongue. Incisures express gap junctions and their contribution to the electrical properties of myelin remain mysterious (Kamasawa et al., 2005). Likewise, exquisite arrangements of structures apposing glial and neuronal membranes and also with other glial membranes in the form of “rosettes” formed by ion channels at the paranode are still poorly understood (Rash et al., 2016).

These findings open up the following question: how do these nanostructures impact function? One would expect that further perturbing and studying those nanoscale structures in the future will further our understanding of how they contribute to axonal computations.

### 2.4. Axons Are Not Alone: Intracellular and Intercellular Coupling

#### 2.4.1. Crosstalk of Axons With Other Compartments

Although we have done a treatment of the axon as an isolated cable as has classically been performed in the early days (Lillie, 1925; Huxley and Stämpfli, 1949; Hodgkin and Huxley, 1952), the axonal cable is coupled to the somatic and dendritic compartment at one end and, additionally, directly or indirectly to electrically-coupled cells, which also functionally act like an electrical compartment (Furshpan and Potter, 1959; Alcami and Marty, 2013; Eyal et al., 2014). Electrical coupling between the axon and the soma, and indirectly to dendrites and electrically-coupled cells, influences AP generation in the axon (Bekkers and Häusser, 2007; Eyal et al., 2014; Amsalem et al., 2016; Alcami, 2018; Goldwyn et al., 2019). Loading of these non-axonal compartments was shown to impact AP threshold and speed (Bekkers and Häusser, 2007; Eyal et al., 2014; Amsalem et al., 2016). Their contribution to the effective membrane time constant is substantial. The somato-dendritic compartment has been shown to, by this mechanism, modify both the threshold and the initial rise of the AP (Eyal et al., 2014).

It is further interesting to consider the axon in the context of synaptic integration, that is, the computation of information received at synapses. Similar to the somato-dendritic and junctional compartments, which act as current sinks, influencing the effective kinetics and strength of excitatory inputs recorded at the soma, before they reach the AIS (Nörenberg et al., 2010; Alcami, 2018), the axonal membrane also behaves as a current sink, leaking current generated at synapses in the somatic and dendritic compartments. This phenomenon has been shown to, through a passive mechanism, accelerate the time-course of somatically-recorded excitatory synaptic events (Mejia-Gervacio et al., 2007). It has additionally been suggested to also accelerate the time-course of spikelets generated by electrically-coupled cells (Alcami and Marty, 2013). Mejia-Gervacio and collaborators (Mejia-Gervacio et al., 2007) show that the capacitive loading of axons introduces in cerebellar molecular layer interneurons a computational time constant of about 3 ms, which is one order of magnitude slower than the faster time constant to load the somatodendritic compartment. This relatively slowly-charging process of the axonal membrane accelerates the decay of excitatory postsynaptic potentials, reducing the time window for AP generation. Therefore, electrical signals that arrive to the axon are not only passively influenced by other compartments, but they also influence passive computation by the non-axonal compartments, as part of a system formed by coupled compartments.

Let us now turn our attention onto the signals propagating in axons as cells receive synaptic events in their somas and dendrites, which is a consequence of the current flow in the axon evoked by synaptic events received in dendrites and somas. These signals propagate at long distances before their complete attenuation (contributing to the analog signaling in the axon that was previously introduced). The space constant at the hippocampal unmyelinated granule cell axon is in the range of hundreds of micrometers (Alle and Geiger, 2006) and the subthreshold propagation of voltage depolarizations has been shown to increase AP evoked release (Shu et al., 2006). Interestingly, subthreshold signals do not only travel orthodromically toward the terminals, but signals generated in the axon, e.g., by synaptic receptors present on the presynaptic membrane, also travel antidromically to the soma (Trigo et al., 2010). As a consequence, these axonal events depolarize the soma and influence AP generation (de San Martin et al., 2015). In summary, intracellular and intercellular coupling render the computations of all compartments inter-dependent: axons don't carry out their computations independently from other compartments of the same cell or from other cells, and non-axonal compartments don't carry their computations independently of axons.

#### 2.4.2. Direct Coupling Between Axons

**Figure 6:**
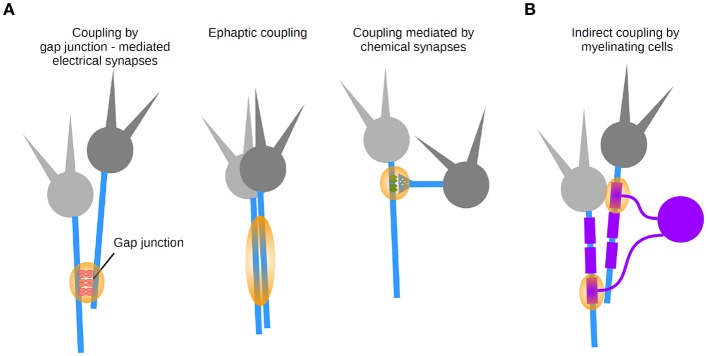
Coupling modalities between axons. **(A)** Three types of synapse-mediated coupling: through axo-axonal gap junctions (left), ephaptic coupling (middle), and chemical axonic synapses (right). **(B)** Indirect coupling via myelinating cells.

Direct coupling between axons was suggested in early work, bringing up the concept that networks of axons may directly interact with each other (Katz and Schmitt, 1940). These interactions can be explained by two forms of electrical transmission (Figure 6A): ephaptic transmission due to the generation of an electric field by an axon, affecting the excitability of a neighboring axon (Katz and Schmitt, 1940; Blot and Barbour, 2014; Han et al., 2018), and transmission mediated by gap junction-mediated electrical synapses (Furshpan and Potter, 1959; Watanabe and Grundfest, 1961; Bennett et al., 1963; Robertson et al., 1963; Schmitz et al., 2001). As a matter of fact, electrical synapses were first discovered in axons in a variety of preparations (Furshpan and Potter, 1959; Watanabe and Grundfest, 1961; Bennett et al., 1963; Robertson et al., 1963).

Axonal electrical synapses are expected to strongly affect electrical signals by adding a conductance pathway directly in the axon. Remarkably, axonal gap junctions allow inputs to arrive at the axon directly, blurring the pure “output” role of axons: an AP in a presynaptic axon induces a spikelet (a low-pass filtered version of the presynaptic AP) in the postsynaptic axon. Spikelets excite distal parts of the axon where the gap junctions are located. When the spikelet depolarizes the membrane potential sufficiently, they are able to evoke APs (Chorev and Brecht, 2012). Interestingly, *in vivo* recordings from hippocampal principal cells in CA regions from rodents revealed two types of APs: one type was preceded by a “shoulder” which was identical to the rising phase of the spikelet, and the other was a full-blown AP lacking the shoulder (Epsztein et al., 2010). Spikelets correlate with AP firing from nearby cells, confirming that they are generated by electrically-coupled cells, and not by spontaneous AP firing of distal axons (Chorev and Brecht, 2012). Altogether, these studies suggest that hippocampal pyramidal cell axons are excited through electrical synapses under physiological conditions in behaving animals, as it had been previously shown in slices (Schmitz et al., 2001).

Axonal gap junctions have additionally been proposed to underlie fast synchronization of neuronal ensembles in both physiological and pathophysiological conditions (Schmitz et al., 2001; Roopun et al., 2010). Interestingly, axo-axonal gap junctions can have a strong impact on spike coordination and coding efficiency (Wang et al., 2017). They may additionally induce AP propagation failures in thin axons as shown in a model of a brainstem networks in frog tadpoles (Hull et al., 2015). In this model, gap junction-mediated failures can be prevented by increasing gap junction resistance or membrane excitability.

Last but not least, direct axo-axonal coupling can be mediated by chemical synapses onto axons (Figure 6A) which are typically performed by a specific type of interneuron targeting the AIS of principal cells (chandelier cells in the neocortex; Somogyi et al., 1985). GABAergic terminals made onto neocortical and hippocampal pyramidal cells axons exert inhibitory control over AP initiation (Dugladze et al., 2012). Owing to axo-axonal coupling, populations of axons can be regarded as a computational unit.

#### 2.4.3. Axons Interact Indirectly via Glial Cells

Finally, let us consider the contribution of glial cells to axonal function as an indirect coupling pathway between axons (Figure 6B). The biophysical properties of myelin are achieved by glial cells that produce myelin. Since several axons are typically contacted by a myelinating oligodendrocyte, this introduces de facto an indirect coupling pathway between axons. A number of transmission mechanisms has been described between axons and myelin (reviewed in Micu et al., 2017), whose dynamic properties depend on signaling from neurons (Hines et al., 2015). A better understanding of axo-axonal coupling mediated by glial cells will in the long run help us understand the computations of populations of axons regulated by glial cells in health and disease.

## 3. Overview of Axonal Computations

Let us at this point recall the definition of computation stated beforehand. We defined computations as modifications in the input/output transformations performed by axons. In this section, we will summarize the diverse axonal computations afforded by the various physiological processes discussed throughout the article beside the well established canonical computation of action potential generation at the AIS.

1. Axons compute with both analog and digital signals. This can also be termed “hybrid computation” since both signals co-exist and are tightly intermingled.
2. Axons can vary the shape of APs in terms of width and rise time by combining ion channels with specific kinetics and by distributing them in specific spatial configurations along the axon. These variations modulate their postsynaptic impact.
3. Owing to specializations in morphology, active properties of axons or electrical coupling between axons, (i) APs can fail to propagate, (ii) their efficient propagation can be ensured, or (iii) they may reflect to the mother branch. This implies that an AP in the AIS may correspond at the terminal to more than one AP (reflection case), or less than one AP (failure case).
4. By fine-tuning axonal morphology, active and passive mechanisms, axons can introduce anisotropies in AP propagation speed along the axon.
5. Axons modulate synaptic integration by fine-tuning AIS function and by leaking currents in the axonal tree.
6. By generating AIS spikes and ectopic spikes, and propagating them orthodromically and antidromically respectively, axons can carry parallel computations.
7. By virtue of their plasticity, axons and myelin readjust AP propagation speed.
8. Axons can add and relocate computational “hotspots” that generate non-linear transformations at specialized locations: AIS, nodes, ectopic spike generation sites, heminode, branching points, daughter branches, terminals.
9. Axons can compute information at the level of populations of axons owing to the various modalities of inter-axonal coupling.
10. Axon can, in addition to their output role, act as input structures by receiving information at electrical synapses, chemical axo-axonic synapses, and axonal receptors.

## 4. Conclusion

Here, we have reviewed the biophysical nature of computations performed by the axon. We have shown that the axon is not just a cable on which electrical pulses propagate but rather a computational device that modulates signaling and adds to the complexity of information processing in the brain. It should be appreciated that these computations are sophisticated enough to contribute to network level phenomena up to behavior *in vivo*. Relating axonal computation to behavioral phenomenology is still a nascent area which will complement studies that have focused almost exclusively on somas, dendrites or a coarse grained view of neurons. The study of axonal computation is hurdled by technical challenges, but there is already an emerging interest in developing technologies that will allow to electrophysiologically and structurally study axonal processes. Further complication arises when one realizes that axons do not act alone but in concert, exhibiting collective modes of computation conveyed by inter-axonal signaling. We propose that axonal biophysics are of vital importance for understanding not only how single neurons process information but also how neural networks coordinate their activity.

## Author Contributions

All authors listed have made a substantial, direct and intellectual contribution to the work, and approved it for publication.

### Conflict of Interest Statement

The authors declare that the research was conducted in the absence of any commercial or financial relationships that could be construed as a potential conflict of interest.
